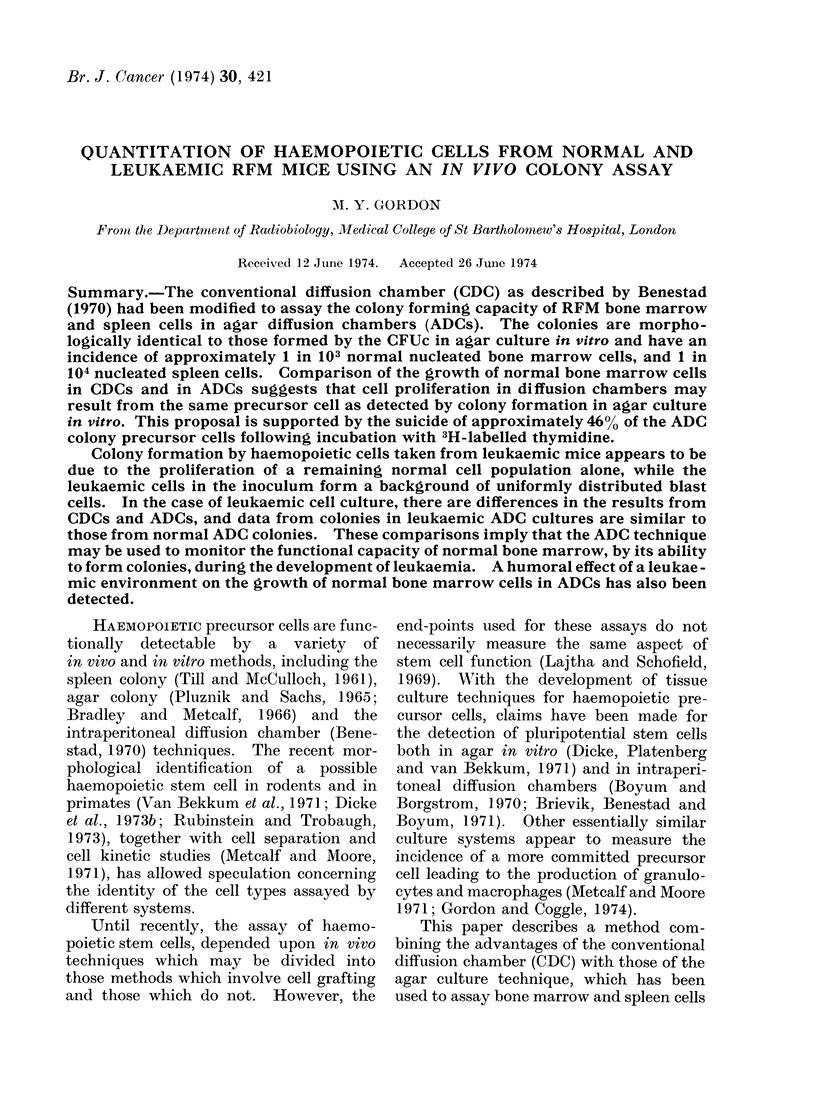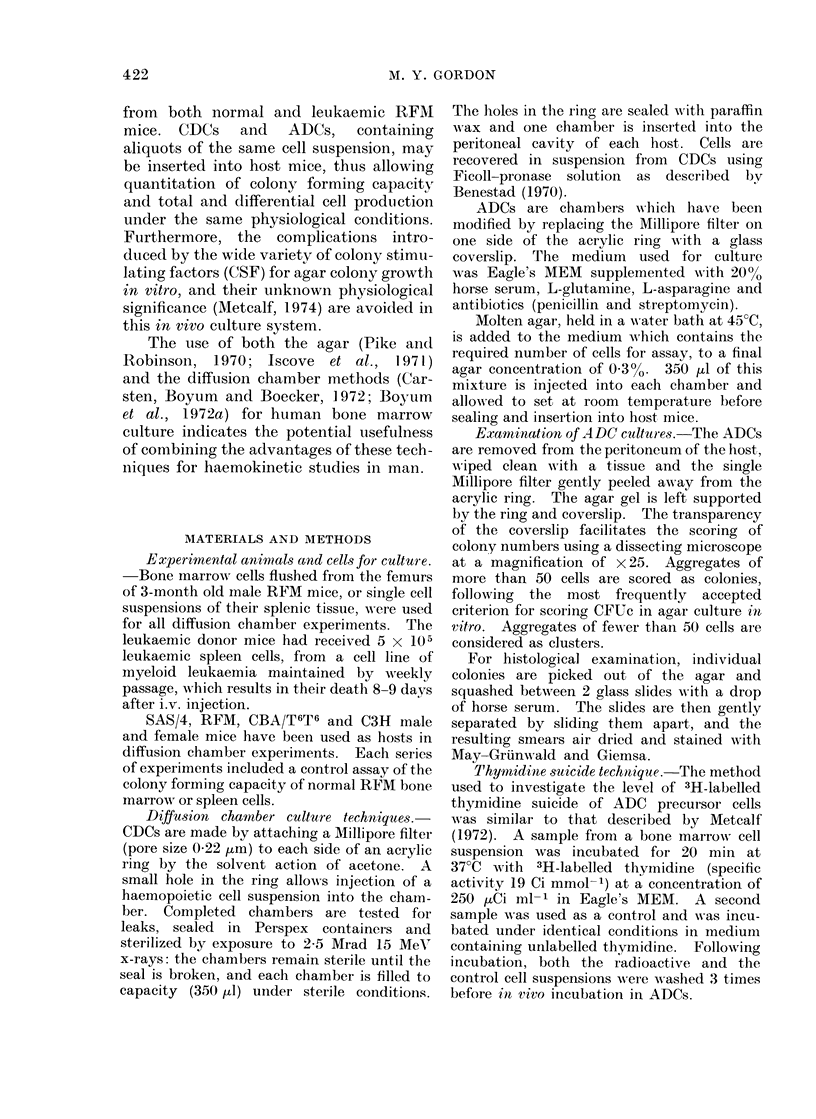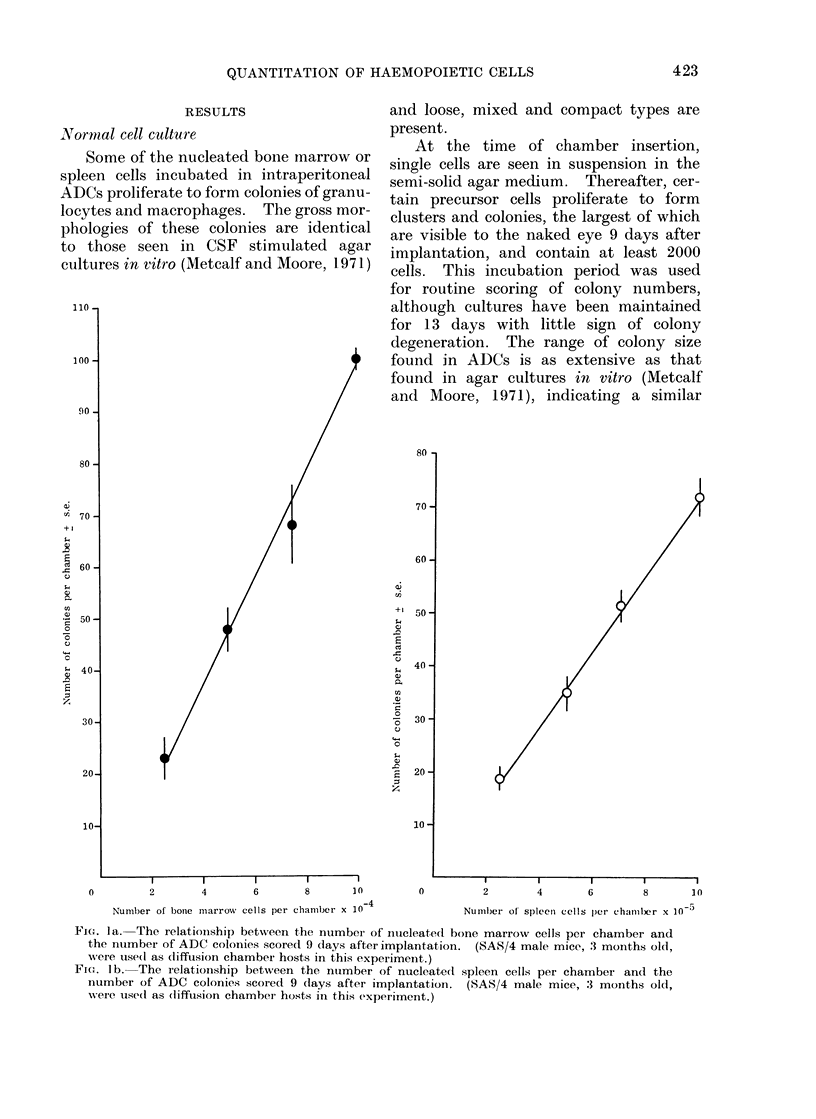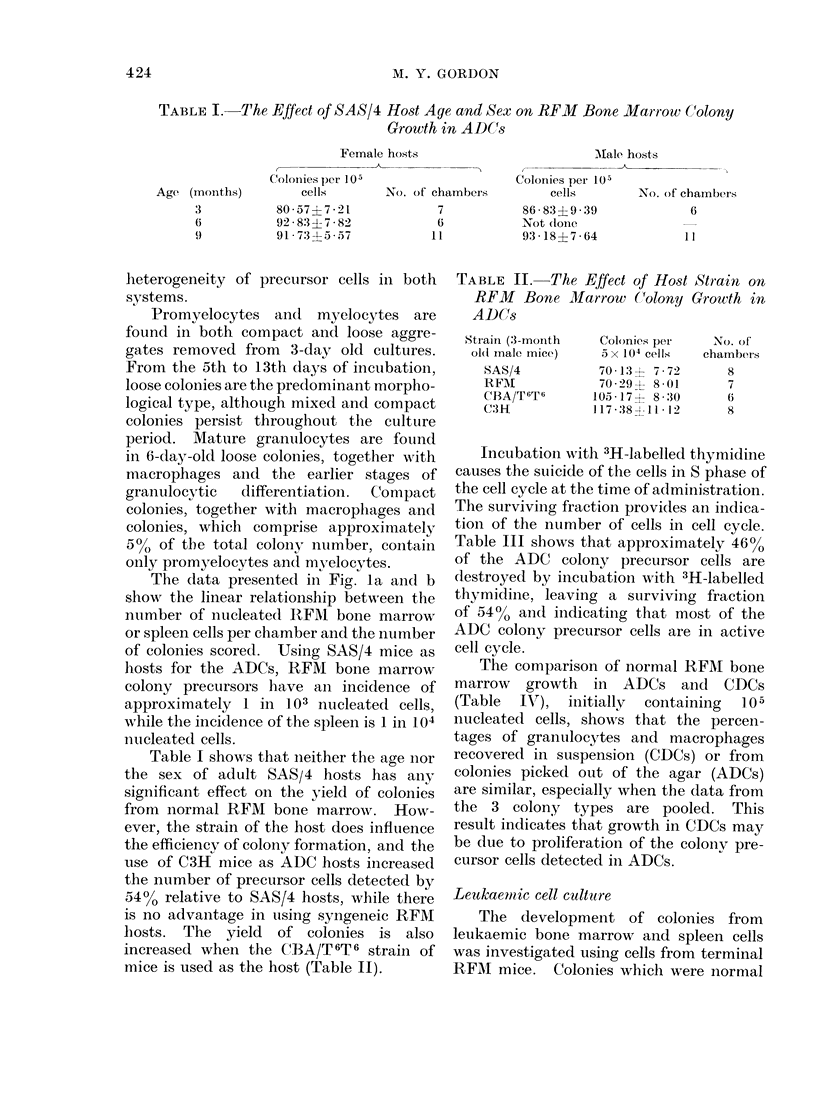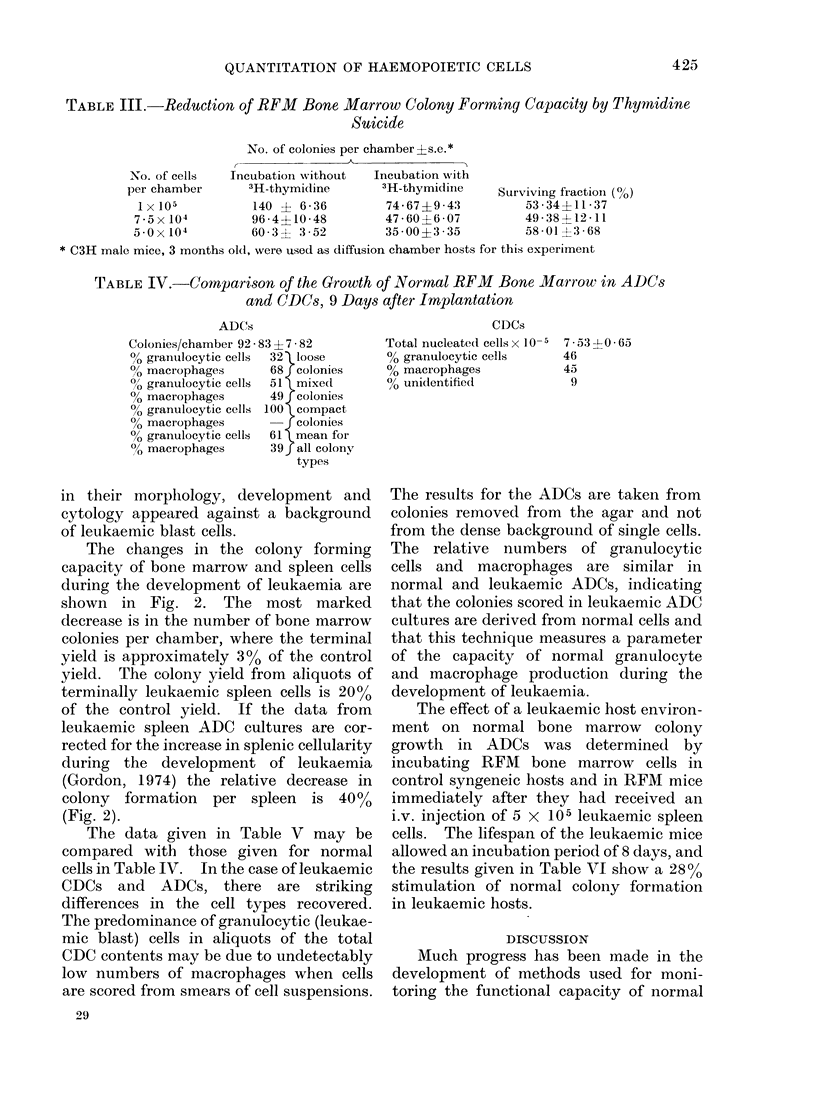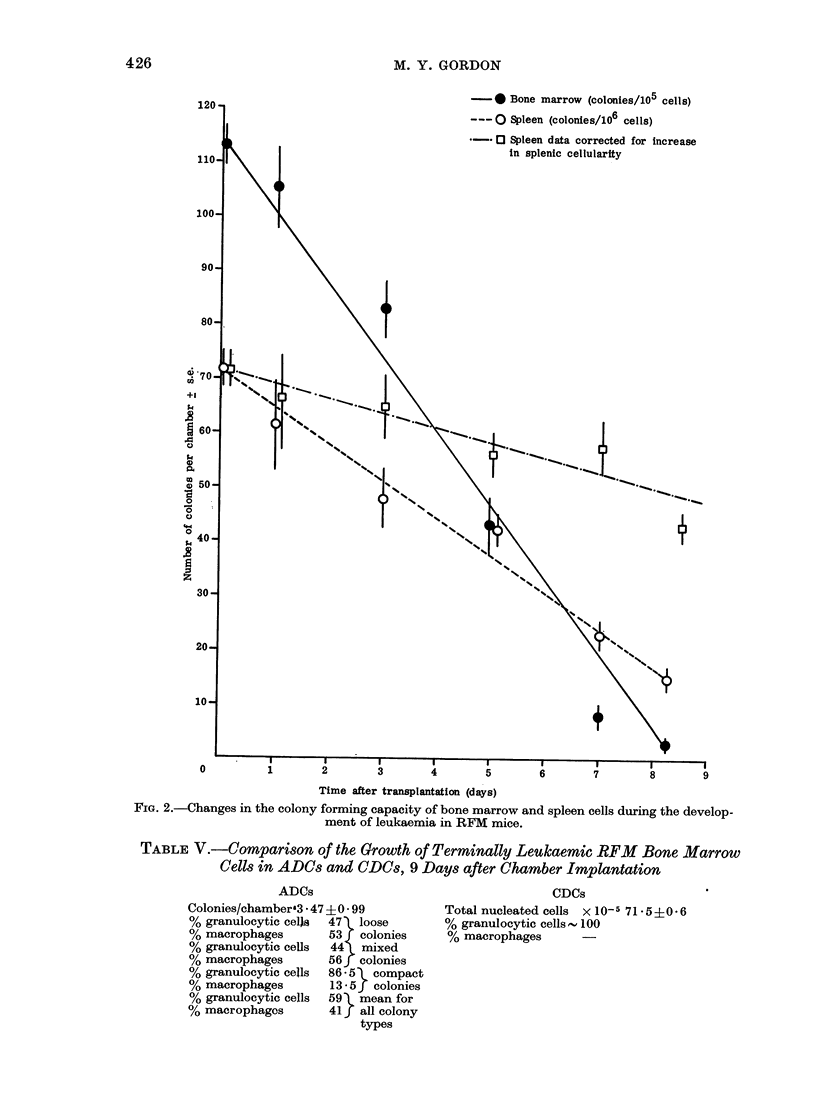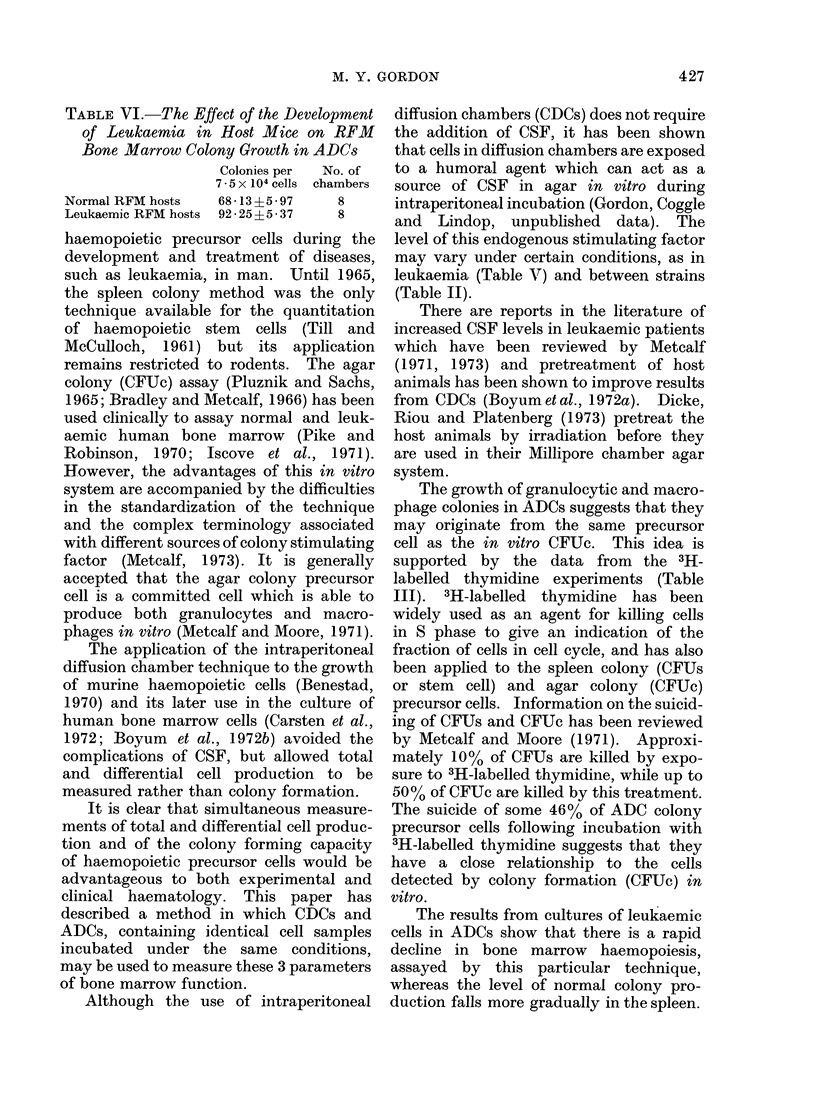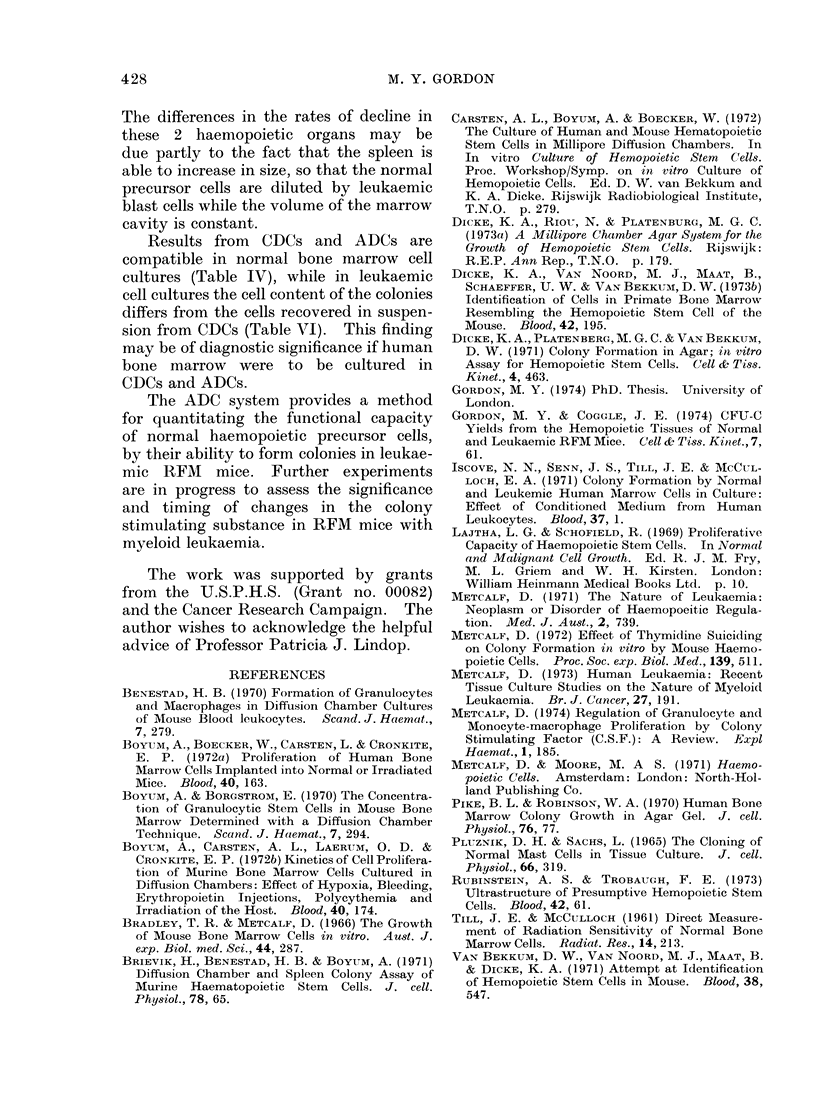# Quantitation of Haemopoietic Cells from Normal and Leukaemic RFM Mice Using an In Vivo Colony Assay

**DOI:** 10.1038/bjc.1974.216

**Published:** 1974-11

**Authors:** M. Y. Gordon

## Abstract

The conventional diffusion chamber (CDC) as described by Benestad (1970) had been modified to assay the colony forming capacity of RFM bone marrow and spleen cells in agar diffusion chambers (ADCs). The colonies are morphologically identical to those formed by the CFUc in agar culture *in vitro* and have an incidence of approximately 1 in 10^3^ normal nucleated bone marrow cells, and 1 in 10^4^ nucleated spleen cells. Comparison of the growth of normal bone marrow cells in CDCs and in ADCs suggests that cell proliferation in diffusion chambers may result from the same precursor cell as detected by colony formation in agar culture *in vitro.* This proposal is supported by the suicide of approximately 46% of the ADC colony precursor cells following incubation with ^3^H-labelled thymidine.

Colony formation by haemopoietic cells taken from leukaemic mice appears to be due to the proliferation of a remaining normal cell population alone, while the leukaemic cells in the inoculum form a background of uniformly distributed blast cells. In the case of leukaemic cell culture, there are differences in the results from CDCs and ADCs, and data from colonies in leukaemic ADC cultures are similar to those from normal ADC colonies. These comparisons imply that the ADC technique may be used to monitor the functional capacity of normal bone marrow, by its ability to form colonies, during the development of leukaemia. A humoral effect of a leukaemic environment on the growth of normal bone marrow cells in ADCs has also been detected.


*Br. J. Cancer* (1974) **30**, 421

### QUANTITATION OF HAEMOPOIETIC CELLS FROM NORMAL AND LEUKAEMIC RFM MICE USING AN *IN VIVO* COLONY ASSAY

M. Y. GORDON

*From the Department of Radiobiology, Medical College of St Bartholomew's Hospital, London*

Received 12 June 1974. Accepted 26 June 1974

**Summary.—The conventional diffusion chamber (CDC) as described by Benestad (1970) had been modified to assay the colony forming capacity of RFM bone marrow and spleen cells in agar diffusion chambers (ADCs). The colonies are morphologically identical to those formed by the CFUc in agar culture *in vitro* and have an incidence of approximately 1 in $10^3$ normal nucleated bone marrow cells, and 1 in $10^4$ nucleated spleen cells. Comparison of the growth of normal bone marrow cells in CDCs and in ADCs suggests that cell proliferation in diffusion chambers may result from the same precursor cell as detected by colony formation in agar culture *in vitro*. This proposal is supported by the suicide of approximately 46% of the ADC colony precursor cells following incubation with $^3$H-labelled thymidine.**

**Colony formation by haemopoietic cells taken from leukaemic mice appears to be due to the proliferation of a remaining normal cell population alone, while the leukaemic cells in the inoculum form a background of uniformly distributed blast cells. In the case of leukaemic cell culture, there are differences in the results from CDCs and ADCs, and data from colonies in leukaemic ADC cultures are similar to those from normal ADC colonies. These comparisons imply that the ADC technique may be used to monitor the functional capacity of normal bone marrow, by its ability to form colonies, during the development of leukaemia. A humoral effect of a leukaemic environment on the growth of normal bone marrow cells in ADCs has also been detected.**

HAEMOPOIETIC precursor cells are functionally detectable by a variety of *in vivo* and *in vitro* methods, including the spleen colony (Till and McCulloch, 1961), agar colony (Pluznik and Sachs, 1965; Bradley and Metcalf, 1966) and the intraperitoneal diffusion chamber (Benestad, 1970) techniques. The recent morphological identification of a possible haemopoietic stem cell in rodents and in primates (Van Bekkum *et al.*, 1971; Dicke *et al.*, 1973*b*; Rubinstein and Trobaugh, 1973), together with cell separation and cell kinetic studies (Metcalf and Moore, 1971), has allowed speculation concerning the identity of the cell types assayed by different systems.

Until recently, the assay of haemopoietic stem cells, depended upon *in vivo* techniques which may be divided into those methods which involve cell grafting and those which do not. However, the end-points used for these assays do not necessarily measure the same aspect of stem cell function (Lajtha and Schofield, 1969). With the development of tissue culture techniques for haemopoietic precursor cells, claims have been made for the detection of pluripotential stem cells both in agar *in vitro* (Dicke, Platenberg and van Bekkum, 1971) and in intraperitoneal diffusion chambers (Boyum and Borgstrom, 1970; Brievik, Benestad and Boyum, 1971). Other essentially similar culture systems appear to measure the incidence of a more committed precursor cell leading to the production of granulocytes and macrophages (Metcalf and Moore 1971; Gordon and Coggle, 1974).

This paper describes a method combining the advantages of the conventional diffusion chamber (CDC) with those of the agar culture technique, which has been used to assay bone marrow and spleen cells from both normal and leukaemic RFM mice. CDCs and ADCs, containing aliquots of the same cell suspension, may be inserted into host mice, thus allowing quantitation of colony forming capacity and total and differential cell production under the same physiological conditions. Furthermore, the complications introduced by the wide variety of colony stimulating factors (CSF) for agar colony growth *in vitro*, and their unknown physiological significance (Metcalf, 1974) are avoided in this *in vivo* culture system.

The use of both the agar (Pike and Robinson, 1970; Iscove *et al.*, 1971) and the diffusion chamber methods (Carsten, Boyum and Boecker, 1972; Boyum *et al.*, 1972*a*) for human bone marrow culture indicates the potential usefulness of combining the advantages of these techniques for haemokinetic studies in man.

### MATERIALS AND METHODS

*Experimental animals and cells for culture.*—Bone marrow cells flushed from the femurs of 3-month old male RFM mice, or single cell suspensions of their splenic tissue, were used for all diffusion chamber experiments. The leukaemic donor mice had received $5 \times 10^5$ leukaemic spleen cells, from a cell line of myeloid leukaemia maintained by weekly passage, which results in their death 8–9 days after i.v. injection.

SAS/4, RFM, CBA/T$^6$T$^6$ and C3H male and female mice have been used as hosts in diffusion chamber experiments. Each series of experiments included a control assay of the colony forming capacity of normal RFM bone marrow or spleen cells.

*Diffusion chamber culture techniques.*—CDCs are made by attaching a Millipore filter (pore size 0·22 $\mu$m) to each side of an acrylic ring by the solvent action of acetone. A small hole in the ring allows injection of a haemopoietic cell suspension into the chamber. Completed chambers are tested for leaks, sealed in Perspex containers and sterilized by exposure to 2·5 Mrad 15 MeV x-rays: the chambers remain sterile until the seal is broken, and each chamber is filled to capacity (350 $\mu$l) under sterile conditions. The holes in the ring are sealed with paraffin wax and one chamber is inserted into the peritoneal cavity of each host. Cells are recovered in suspension from CDCs using Ficoll–pronase solution as described by Benestad (1970).

ADCs are chambers which have been modified by replacing the Millipore filter on one side of the acrylic ring with a glass coverslip. The medium used for culture was Eagle's MEM supplemented with 20% horse serum, L-glutamine, L-asparagine and antibiotics (penicillin and streptomycin).

Molten agar, held in a water bath at 45°C, is added to the medium which contains the required number of cells for assay, to a final agar concentration of 0·3%. 350 $\mu$l of this mixture is injected into each chamber and allowed to set at room temperature before sealing and insertion into host mice.

*Examination of ADC cultures.*—The ADCs are removed from the peritoneum of the host, wiped clean with a tissue and the single Millipore filter gently peeled away from the acrylic ring. The agar gel is left supported by the ring and coverslip. The transparency of the coverslip facilitates the scoring of colony numbers using a dissecting microscope at a magnification of $\times 25$. Aggregates of more than 50 cells are scored as colonies, following the most frequently accepted criterion for scoring CFUc in agar culture *in vitro*. Aggregates of fewer than 50 cells are considered as clusters.

For histological examination, individual colonies are picked out of the agar and squashed between 2 glass slides with a drop of horse serum. The slides are then gently separated by sliding them apart, and the resulting smears air dried and stained with May–Grünwald and Giemsa.

*Thymidine suicide technique.*—The method used to investigate the level of $^3$H-labelled thymidine suicide of ADC precursor cells was similar to that described by Metcalf (1972). A sample from a bone marrow cell suspension was incubated for 20 min at 37°C with $^3$H-labelled thymidine (specific activity 19 Ci mmol$^{-1}$) at a concentration of 250 $\mu$Ci ml$^{-1}$ in Eagle's MEM. A second sample was used as a control and was incubated under identical conditions in medium containing unlabelled thymidine. Following incubation, both the radioactive and the control cell suspensions were washed 3 times before *in vivo* incubation in ADCs.

### RESULTS

### *Normal cell culture*

Some of the nucleated bone marrow or spleen cells incubated in intraperitoneal ADCs proliferate to form colonies of granulocytes and macrophages. The gross morphologies of these colonies are identical to those seen in CSF stimulated agar cultures *in vitro* (Metcalf and Moore, 1971) and loose, mixed and compact types are present.

At the time of chamber insertion, single cells are seen in suspension in the semi-solid agar medium. Thereafter, certain precursor cells proliferate to form clusters and colonies, the largest of which are visible to the naked eye 9 days after implantation, and contain at least 2000 cells. This incubation period was used for routine scoring of colony numbers, although cultures have been maintained for 13 days with little sign of colony degeneration. The range of colony size found in ADCs is as extensive as that found in agar cultures *in vitro* (Metcalf and Moore, 1971), indicating a similar

FIG. 1a.—The relationship between the number of nucleated bone marrow cells per chamber and the number of ADC colonies scored 9 days after implantation. (SAS/4 male mice, 3 months old, were used as diffusion chamber hosts in this experiment.)

FIG. 1b.—The relationship between the number of nucleated spleen cells per chamber and the number of ADC colonies scored 9 days after implantation. (SAS/4 male mice, 3 months old, were used as diffusion chamber hosts in this experiment.)

TABLE I.—*The Effect of SAS/4 Host Age and Sex on RFM Bone Marrow Colony Growth in ADCs*

| | Female hosts | | Male hosts | |
|---|---|---|---|---|
| Age (months) | Colonies per $10^5$ cells | No. of chambers | Colonies per $10^5$ cells | No. of chambers |
| 3 | 80·57±7·21 | 7 | 86·83±9·39 | 6 |
| 6 | 92·83±7·82 | 6 | Not done | — |
| 9 | 91·73±5·57 | 11 | 93·18±7·64 | 11 |

heterogeneity of precursor cells in both systems.

Promyelocytes and myelocytes are found in both compact and loose aggregates removed from 3-day old cultures. From the 5th to 13th days of incubation, loose colonies are the predominant morphological type, although mixed and compact colonies persist throughout the culture period. Mature granulocytes are found in 6-day-old loose colonies, together with macrophages and the earlier stages of granulocytic differentiation. Compact colonies, together with macrophages and colonies, which comprise approximately 5% of the total colony number, contain only promyelocytes and myelocytes.

The data presented in Fig. 1a and b show the linear relationship between the number of nucleated RFM bone marrow or spleen cells per chamber and the number of colonies scored. Using SAS/4 mice as hosts for the ADCs, RFM bone marrow colony precursors have an incidence of approximately 1 in $10^3$ nucleated cells, while the incidence of the spleen is 1 in $10^4$ nucleated cells.

Table I shows that neither the age nor the sex of adult SAS/4 hosts has any significant effect on the yield of colonies from normal RFM bone marrow. However, the strain of the host does influence the efficiency of colony formation, and the use of C3H mice as ADC hosts increased the number of precursor cells detected by 54% relative to SAS/4 hosts, while there is no advantage in using syngeneic RFM hosts. The yield of colonies is also increased when the CBA/$T^6T^6$ strain of mice is used as the host (Table II).

TABLE II.—*The Effect of Host Strain on RFM Bone Marrow Colony Growth in ADCs*

| Strain (3-month old male mice) | Colonies per $5\times10^4$ cells | No. of chambers |
|---|---|---|
| SAS/4 | 70·13± 7·72 | 8 |
| RFM | 70·29± 8·01 | 7 |
| CBA/$T^6T^6$ | 105·17± 8·30 | 6 |
| C3H | 117·38±11·12 | 8 |

Incubation with $^3$H-labelled thymidine causes the suicide of the cells in S phase of the cell cycle at the time of administration. The surviving fraction provides an indication of the number of cells in cell cycle. Table III shows that approximately 46% of the ADC colony precursor cells are destroyed by incubation with $^3$H-labelled thymidine, leaving a surviving fraction of 54% and indicating that most of the ADC colony precursor cells are in active cell cycle.

The comparison of normal RFM bone marrow growth in ADCs and CDCs (Table IV), initially containing $10^5$ nucleated cells, shows that the percentages of granulocytes and macrophages recovered in suspension (CDCs) or from colonies picked out of the agar (ADCs) are similar, especially when the data from the 3 colony types are pooled. This result indicates that growth in CDCs may be due to proliferation of the colony precursor cells detected in ADCs.

*Leukaemic cell culture*

The development of colonies from leukaemic bone marrow and spleen cells was investigated using cells from terminal RFM mice. Colonies which were normal

TABLE III.—*Reduction of RFM Bone Marrow Colony Forming Capacity by Thymidine Suicide*

| No. of cells per chamber | No. of colonies per chamber ± s.e.* Incubation without $^3$H-thymidine | Incubation with $^3$H-thymidine | Surviving fraction (%) |
|---|---|---|---|
| $1\times10^5$ | 140 ± 6·36 | 74·67 ± 9·43 | 53·34 ± 11·37 |
| $7{\cdot}5\times10^4$ | 96·4 ± 10·48 | 47·60 ± 6·07 | 49·38 ± 12·11 |
| $5{\cdot}0\times10^4$ | 60·3 ± 3·52 | 35·00 ± 3·35 | 58·01 ± 3·68 |

* C3H male mice, 3 months old, were used as diffusion chamber hosts for this experiment

TABLE IV.—*Comparison of the Growth of Normal RFM Bone Marrow in ADCs and CDCs, 9 Days after Implantation*

ADCs

| | | |
|---|---|---|
| Colonies/chamber | 92·83 ± 7·82 | |
| % granulocytic cells | 32 | loose colonies |
| % macrophages | 68 | |
| % granulocytic cells | 51 | mixed colonies |
| % macrophages | 49 | |
| % granulocytic cells | 100 | compact colonies |
| % macrophages | — | |
| % granulocytic cells | 61 | mean for all colony types |
| % macrophages | 39 | |

CDCs

| | |
|---|---|
| Total nucleated cells $\times10^{-5}$ | 7·53 ± 0·65 |
| % granulocytic cells | 46 |
| % macrophages | 45 |
| % unidentified | 9 |

in their morphology, development and cytology appeared against a background of leukaemic blast cells.

The changes in the colony forming capacity of bone marrow and spleen cells during the development of leukaemia are shown in Fig. 2. The most marked decrease is in the number of bone marrow colonies per chamber, where the terminal yield is approximately 3% of the control yield. The colony yield from aliquots of terminally leukaemic spleen cells is 20% of the control yield. If the data from leukaemic spleen ADC cultures are corrected for the increase in splenic cellularity during the development of leukaemia (Gordon, 1974) the relative decrease in colony formation per spleen is 40% (Fig. 2).

The data given in Table V may be compared with those given for normal cells in Table IV. In the case of leukaemic CDCs and ADCs, there are striking differences in the cell types recovered. The predominance of granulocytic (leukaemic blast) cells in aliquots of the total CDC contents may be due to undetectably low numbers of macrophages when cells are scored from smears of cell suspensions. The results for the ADCs are taken from colonies removed from the agar and not from the dense background of single cells. The relative numbers of granulocytic cells and macrophages are similar in normal and leukaemic ADCs, indicating that the colonies scored in leukaemic ADC cultures are derived from normal cells and that this technique measures a parameter of the capacity of normal granulocyte and macrophage production during the development of leukaemia.

The effect of a leukaemic host environment on normal bone marrow colony growth in ADCs was determined by incubating RFM bone marrow cells in control syngeneic hosts and in RFM mice immediately after they had received an i.v. injection of $5\times10^5$ leukaemic spleen cells. The lifespan of the leukaemic mice allowed an incubation period of 8 days, and the results given in Table VI show a 28% stimulation of normal colony formation in leukaemic hosts.

### DISCUSSION

Much progress has been made in the development of methods used for monitoring the functional capacity of normal

FIG. 2.—Changes in the colony forming capacity of bone marrow and spleen cells during the development of leukaemia in RFM mice.

TABLE V.—*Comparison of the Growth of Terminally Leukaemic RFM Bone Marrow Cells in ADCs and CDCs, 9 Days after Chamber Implantation*

| ADCs | | | CDCs | |
|---|---|---|---|---|
| Colonies/chamber | 3·47±0·99 | | Total nucleated cells $\times 10^{-5}$ | 71·5±0·6 |
| % granulocytic cells | 47 | loose colonies | % granulocytic cells | ~100 |
| % macrophages | 53 | | % macrophages | — |
| % granulocytic cells | 44 | mixed colonies | | |
| % macrophages | 56 | | | |
| % granulocytic cells | 86·5 | compact colonies | | |
| % macrophages | 13·5 | | | |
| % granulocytic cells | 59 | mean for all colony types | | |
| % macrophages | 41 | | | |

TABLE VI.—*The Effect of the Development of Leukaemia in Host Mice on RFM Bone Marrow Colony Growth in ADCs*

| | Colonies per $7 \cdot 5 \times 10^4$ cells | No. of chambers |
|---|---|---|
| Normal RFM hosts | $68 \cdot 13 \pm 5 \cdot 97$ | 8 |
| Leukaemic RFM hosts | $92 \cdot 25 \pm 5 \cdot 37$ | 8 |

haemopoietic precursor cells during the development and treatment of diseases, such as leukaemia, in man. Until 1965, the spleen colony method was the only technique available for the quantitation of haemopoietic stem cells (Till and McCulloch, 1961) but its application remains restricted to rodents. The agar colony (CFUc) assay (Pluznik and Sachs, 1965; Bradley and Metcalf, 1966) has been used clinically to assay normal and leukaemic human bone marrow (Pike and Robinson, 1970; Iscove *et al.*, 1971). However, the advantages of this *in vitro* system are accompanied by the difficulties in the standardization of the technique and the complex terminology associated with different sources of colony stimulating factor (Metcalf, 1973). It is generally accepted that the agar colony precursor cell is a committed cell which is able to produce both granulocytes and macrophages *in vitro* (Metcalf and Moore, 1971).

The application of the intraperitoneal diffusion chamber technique to the growth of murine haemopoietic cells (Benestad, 1970) and its later use in the culture of human bone marrow cells (Carsten *et al.*, 1972; Boyum *et al.*, 1972*b*) avoided the complications of CSF, but allowed total and differential cell production to be measured rather than colony formation.

It is clear that simultaneous measurements of total and differential cell production and of the colony forming capacity of haemopoietic precursor cells would be advantageous to both experimental and clinical haematology. This paper has described a method in which CDCs and ADCs, containing identical cell samples incubated under the same conditions, may be used to measure these 3 parameters of bone marrow function.

Although the use of intraperitoneal diffusion chambers (CDCs) does not require the addition of CSF, it has been shown that cells in diffusion chambers are exposed to a humoral agent which can act as a source of CSF in agar *in vitro* during intraperitoneal incubation (Gordon, Coggle and Lindop, unpublished data). The level of this endogenous stimulating factor may vary under certain conditions, as in leukaemia (Table V) and between strains (Table II).

There are reports in the literature of increased CSF levels in leukaemic patients which have been reviewed by Metcalf (1971, 1973) and pretreatment of host animals has been shown to improve results from CDCs (Boyum *et al.*, 1972*a*). Dicke, Riou and Platenberg (1973) pretreat the host animals by irradiation before they are used in their Millipore chamber agar system.

The growth of granulocytic and macrophage colonies in ADCs suggests that they may originate from the same precursor cell as the *in vitro* CFUc. This idea is supported by the data from the $^3$H-labelled thymidine experiments (Table III). $^3$H-labelled thymidine has been widely used as an agent for killing cells in S phase to give an indication of the fraction of cells in cell cycle, and has also been applied to the spleen colony (CFUs or stem cell) and agar colony (CFUc) precursor cells. Information on the suiciding of CFUs and CFUc has been reviewed by Metcalf and Moore (1971). Approximately 10% of CFUs are killed by exposure to $^3$H-labelled thymidine, while up to 50% of CFUc are killed by this treatment. The suicide of some 46% of ADC colony precursor cells following incubation with $^3$H-labelled thymidine suggests that they have a close relationship to the cells detected by colony formation (CFUc) *in vitro*.

The results from cultures of leukaemic cells in ADCs show that there is a rapid decline in bone marrow haemopoiesis, assayed by this particular technique, whereas the level of normal colony production falls more gradually in the spleen.

The differences in the rates of decline in these 2 haemopoietic organs may be due partly to the fact that the spleen is able to increase in size, so that the normal precursor cells are diluted by leukaemic blast cells while the volume of the marrow cavity is constant.

Results from CDCs and ADCs are compatible in normal bone marrow cell cultures (Table IV), while in leukaemic cell cultures the cell content of the colonies differs from the cells recovered in suspension from CDCs (Table VI). This finding may be of diagnostic significance if human bone marrow were to be cultured in CDCs and ADCs.

The ADC system provides a method for quantitating the functional capacity of normal haemopoietic precursor cells, by their ability to form colonies in leukaemic RFM mice. Further experiments are in progress to assess the significance and timing of changes in the colony stimulating substance in RFM mice with myeloid leukaemia.

The work was supported by grants from the U.S.P.H.S. (Grant no. 00082) and the Cancer Research Campaign. The author wishes to acknowledge the helpful advice of Professor Patricia J. Lindop.

### REFERENCES

BENESTAD, H. B. (1970) Formation of Granulocytes and Macrophages in Diffusion Chamber Cultures of Mouse Blood leukocytes. *Scand. J. Haemat.*, **7**, 279.

BOYUM, A., BOECKER, W., CARSTEN, L. & CRONKITE, E. P. (1972*a*) Proliferation of Human Bone Marrow Cells Implanted into Normal or Irradiated Mice. *Blood*, **40**, 163.

BOYUM, A. & BORGSTROM, E. (1970) The Concentration of Granulocytic Stem Cells in Mouse Bone Marrow Determined with a Diffusion Chamber Technique. *Scand. J. Haemat.*, **7**, 294.

BOYUM, A., CARSTEN, A. L., LAERUM, O. D. & CRONKITE, E. P. (1972*b*) Kinetics of Cell Proliferation of Murine Bone Marrow Cells Cultured in Diffusion Chambers: Effect of Hypoxia, Bleeding, Erythropoietin Injections, Polycythemia and Irradiation of the Host. *Blood*, **40**, 174.

BRADLEY, T. R. & METCALF, D. (1966) The Growth of Mouse Bone Marrow Cells *in vitro*. *Aust. J. exp. Biol. med. Sci.*, **44**, 287.

BRIEVIK, H., BENESTAD, H. B. & BOYUM, A. (1971) Diffusion Chamber and Spleen Colony Assay of Murine Haematopoietic Stem Cells. *J. cell. Physiol.*, **78**, 65.

CARSTEN, A. L., BOYUM, A. & BOECKER, W. (1972) The Culture of Human and Mouse Hematopoietic Stem Cells in Millipore Diffusion Chambers. In In vitro *Culture of Hemopoietic Stem Cells*. Proc. Workshop/Symp. on *in vitro* Culture of Hemopoietic Cells. Ed. D. W. van Bekkum and K. A. Dicke. Rijswijk Radiobiological Institute, T.N.O. p. 279.

DICKE, K. A., RIOU, N. & PLATENBURG, M. G. C. (1973*a*) *A Millipore Chamber Agar System for the Growth of Hemopoietic Stem Cells*. Rijswijk: R.E.P. *Ann* Rep., T.N.O. p. 179.

DICKE, K. A., VAN NOORD, M. J., MAAT, B., SCHAEFFER, U. W. & VAN BEKKUM, D. W. (1973*b*) Identification of Cells in Primate Bone Marrow Resembling the Hemopoietic Stem Cell of the Mouse. *Blood*, **42**, 195.

DICKE, K. A., PLATENBERG, M. G. C. & VAN BEKKUM, D. W. (1971) Colony Formation in Agar; *in vitro* Assay for Hemopoietic Stem Cells. *Cell & Tiss. Kinet.*, **4**, 463.

GORDON, M. Y. (1974) PhD. Thesis. University of London.

GORDON, M. Y. & COGGLE, J. E. (1974) CFU-C Yields from the Hemopoietic Tissues of Normal and Leukaemic RFM Mice. *Cell & Tiss. Kinet.*, **7**, 61.

ISCOVE, N. N., SENN, J. S., TILL, J. E. & MCCULLOCH, E. A. (1971) Colony Formation by Normal and Leukemic Human Marrow Cells in Culture: Effect of Conditioned Medium from Human Leukocytes. *Blood*, **37**, 1.

LAJTHA, L. G. & SCHOFIELD, R. (1969) Proliferative Capacity of Haemopoietic Stem Cells. In *Normal and Malignant Cell Growth*. Ed. R. J. M. Fry, M. L. Griem and W. H. Kirsten. London: William Heinmann Medical Books Ltd. p. 10.

METCALF, D. (1971) The Nature of Leukaemia: Neoplasm or Disorder of Haemopoeitic Regulation. *Med. J. Aust.*, **2**, 739.

METCALF, D. (1972) Effect of Thymidine Suiciding on Colony Formation *in vitro* by Mouse Haemopoietic Cells. *Proc. Soc. exp. Biol. Med.*, **139**, 511.

METCALF, D. (1973) Human Leukaemia: Recent Tissue Culture Studies on the Nature of Myeloid Leukaemia. *Br. J. Cancer*, **27**, 191.

METCALF, D. (1974) Regulation of Granulocyte and Monocyte-macrophage Proliferation by Colony Stimulating Factor (C.S.F.): A Review. *Expl Haemat.*, **1**, 185.

METCALF, D. & MOORE, M. A S. (1971) *Haemopoietic Cells*. Amsterdam: London: North-Holland Publishing Co.

PIKE, B. L. & ROBINSON, W. A. (1970) Human Bone Marrow Colony Growth in Agar Gel. *J. cell. Physiol.*, **76**, 77.

PLUZNIK, D. H. & SACHS, L. (1965) The Cloning of Normal Mast Cells in Tissue Culture. *J. cell. Physiol.*, **66**, 319.

RUBINSTEIN, A. S. & TROBAUGH, F. E. (1973) Ultrastructure of Presumptive Hemopoietic Stem Cells. *Blood*, **42**, 61.

TILL, J. E. & MCCULLOCH (1961) Direct Measurement of Radiation Sensitivity of Normal Bone Marrow Cells. *Radiat. Res.*, **14**, 213.

VAN BEKKUM, D. W., VAN NOORD, M. J., MAAT, B. & DICKE, K. A. (1971) Attempt at Identification of Hemopoietic Stem Cells in Mouse. *Blood*, **38**, 547.